# Enhanced sensitivity to optimistic cues is manifested in brain structure: a voxel-based morphometry study

**DOI:** 10.1093/scan/nsab075

**Published:** 2021-06-29

**Authors:** Tatjana Aue, Mihai Dricu, Laura Singh, Dominik A Moser, Raviteja Kotikalapudi

**Affiliations:** Institute of Psychology, University of Bern, Bern 3012, Switzerland; Institute of Psychology, University of Bern, Bern 3012, Switzerland; Institute of Psychology, University of Bern, Bern 3012, Switzerland; Institute of Psychology, University of Bern, Bern 3012, Switzerland; Institute of Psychology, University of Bern, Bern 3012, Switzerland

**Keywords:** expectancy bias, attention bias, optimism robustness, gray matter volume, voxel-based morphometry

## Abstract

Recent research shows that congruent outcomes are more rapidly (and incongruent less rapidly) detected when individuals receive optimistic rather than pessimistic cues, an effect that was termed optimism robustness. In the current voxel-based morphometry study, we examined whether optimism robustness has a counterpart in the brain structure. The participants’ task was to detect two different letters (symbolizing monetary gain or loss) in a visual search matrix. Prior to each onset of the search matrix, two different verbal cues informed our participants about a high probability to gain (optimistic expectancy) or lose (pessimistic expectancy) money. The target presented was either congruent or incongruent with these induced expectancies. Optimism robustness revealed in the participants’ reaction times correlated positively with gray matter volume (GMV) in brain regions involved in selective attention (medial visual association area, intraparietal sulcus), emphasizing the strong intertwinement of optimistic expectancies and attention deployment. In addition, GMV in the primary visual cortex diminished with increasing optimism robustness, in line with the interpretation of optimism robustness arising from a global, context-oriented perception. Future studies should address the malleability of these structural correlates of optimism robustness. Our results may assist in the identification of treatment targets in depression.

## Introduction

Some people remain optimistic even in the face of severe challenges, while others turn toward pessimism and give up. An optimistic outlook ensures motivation and, hence, the pursuit of important goals (Aspinwall and Taylor, 1992; Carver *et al.*, 2010; Dricu, Kress, and Aue, 2020). Beyond its motivational impact, the positive anticipation of one’s future has been shown to accompany mental and physical health (Vickers and Vogeltanz, 2000; Rasmussen *et al.*, 2009; Garrett *et al.*, 2014; Hevey *et al.*, 2014). Thus, investigation of the nature and foundations of robust optimistic future expectancies is strongly indicated.

Optimistic outlooks have been shown to be intimately linked with attention deployment (Segerstrom, 2001; Raila *et al.*, 2015; Peters *et al.*, 2016). Concretely, recent research demonstrated dynamic bidirectional influences between optimistic expectancies and selective attention to positive stimuli. On one hand, an optimistic state may act as a predisposition for a positive attention bias, expressed by attentional shifts to positive cues in the environment. Supportive evidence for causal expectancy influences on attention deployment is revealed in behavioral, functional magnetic resonance imaging (fMRI) and eye tracking data (Kress *et al.*, 2018; Singh *et al.*, 2020). Hence, an optimistic outlook affects how we perceive and attend to our surroundings. On the other hand, selective attention to positive stimuli may, in turn, evoke an optimistic bias (Kress and Aue, 2019). Such a dynamic interplay between optimism and attention bias may, thus, spark an upward spiral of positivity (Garland *et al.*, 2010) that is supportive of mental and physical health.

Notably, individuals appear to be particularly sensitive to optimistic cues in their environment. This claim is based on the observation that optimistic expectancies exert stronger influences on attention deployment than pessimistic expectancies do—a phenomenon that should ultimately stabilize optimistic expectancies and was hence termed optimism robustness (Kress *et al.*, 2018). Specifically, cues suggesting a desirable future outcome (i.e. gain of money) yielded greater reaction time (RT) differences to incongruent *vs* congruent outcomes (i.e. losses *vs* gains) in a visual search task than did cues suggesting undesirable future outcomes (i.e. loss of money; (Kress *et al.*, 2018; Singh *et al.*, 2020)). It therefore appears that the optimism-eliciting cues were more effective in facilitating the detection of rewarding targets (and impeding the detection of punishing targets) than were the pessimism-eliciting cues in facilitating the detection of punishing targets (and impeding the detection of rewarding targets). Furthermore, visual attention (as measured by eye tracking) unfolded somewhat differently for optimistic *vs* pessimistic cues (Kress *et al.*, 2018); Whereas incongruent trials were characterized by slowed initial fixation of the target in the visual search task for both optimistic and pessimistic cues compared with that in congruent trials, this effect was significantly stronger for the optimistic cues.

Moreover, such cue-dependent congruency effects were observed not only in the initial orienting to the target stimuli but also during more controlled attentional processes (i.e. attention maintenance). For instance, individuals maintained their visual attention longer on targets that disconfirmed pessimistic expectancies than on those that disconfirmed optimistic expectancies [(Kress *et al.*, 2018); experiment 2]. Such differential visual attention to incongruent *vs* congruent information may ultimately lead to a robust stabilization of optimistic future expectancies. If undesirable information (e.g. losing money after having expected to gain money) is more readily avoided than desirable information (e.g. gaining money after having expected to lose money), this will induce or increase an already existent bias, shifting an individual’s expectancies continuously to the optimistic direction.

Consistent with this picture, an fMRI study (Singh *et al.*, 2020) revealed stronger neural activity differences between incongruent and congruent trials in the case of optimistic than in the case of pessimistic cues—in particular in the dorsal anterior cingulate cortex (dACC), medial frontal gyrus, medial orbitofrontal cortex and inferior parietal lobule—which is in line with the hypothesis of the brain’s salience and executive control networks mediating causal expectancy influences on visual attention (Kress and Aue, 2017). Notably, these data demonstrate that incongruency leads to especially strong neural processing following optimistic expectancies, suggesting that individuals do not simply ignore information that contradicts their initial optimism. Instead, the ensemble of available fMRI and eye tracking data (Segerstrom, 2001; Peters *et al.*, 2016; Kress *et al.*, 2018; Singh *et al.*, 2020) suggests that the importance of such conflicting evidence is downregulated by withdrawing visual attention from this evidence. Visual avoidance or maintenance would thus function as an emotion regulation strategy [cf. (Aldao *et al.*, 2010; Aue *et al.*, 2013b)].

These observations can be harmonized with research on belief updating (Sharot *et al.*, 2011; Garrett and Sharot, 2014; Kuzmanovic *et al.*, 2015) that revealed the limited capacity of humans to integrate unfavorable new information into future expectancies but easy adjustment of expectancies in response to favorable new information. Specifically, humans are resistant to adapting their future beliefs to information that suggests that they have initially been too optimistic and that they should in fact adapt those beliefs to the pessimistic direction. At the same time, they are willing to update future beliefs when they are confronted with evidence that suggests that they have initially been too pessimistic and are justified in shifting their expectancies to the optimistic direction.

The described findings in the optimism-attention domain (Kress *et al.*, 2018; Singh *et al.*, 2020) may in fact constitute important cognitive mechanisms that support belief updating asymmetries. If participants are presented with new information (represented by the targets in the optimism-attention domain), they can differentially attend to those pieces of information, thereby shaping the updating of their future-directed beliefs (i.e. expectancies). Notably, however, the studied phenomena are not interchangeable. Whereas the phenomena investigated in the current manuscript and earlier studies (Kress *et al.*, 2018; Singh *et al.*, 2020) concern the neurocognitive processes related to attentional processes following the (dis)confirmation of expectancy states (which could be interpreted as representing beliefs), the belief updating the literature considers how those beliefs change when feedback is given. Therefore, the focus of the present study (as in our previous research) lies in the precursors of the belief update phenomenon.

Whereas most participants in previous studies (Kress *et al.*, 2018; Singh *et al.*, 2020) that investigated optimistic and pessimistic expectancy influences on attention deployment were characterized by optimism robustness (i.e. greater sensitivity to optimistic compared with pessimistic cues), there were also individual differences in the strength of optimism robustness displayed. These individual differences may be associated with personality measures of optimism bias [i.e. comparative optimism; (Kress *et al.*, 2018)], pointing to the possibility of a stable trait being at the basis of both comparative optimism and optimism robustness. If optimism robustness is as prominent as prior studies suggest, it may even have structural manifestations in the human brain. Brain anatomy is more stable than neural activity and therefore can be assumed to reflect trait characteristics better than fluctuating neural activity does. It may further inform about the underlying mechanisms of optimism robustness and provide possible treatment targets of related disorders (e.g. depression). Beyond that, the investigation of structural in addition to functional brain correlates of optimism robustness may help in determining similarities and divergences with highly similar constructs such as trait optimism or belief updating. In fact, recent research suggests that such differentiation between concepts is possible. For instance, the investigation of brain anatomy has a considerable diagnostic value in that it can distinguish psychiatric conditions with overlapping behavioral signatures (e.g. schizophrenia and bipolar disorder) and may ultimately enable the determination of the gravity of a psychiatric condition (Scarpazza and De Simone, 2016).

In the current investigation, we hence tested whether brain anatomy is associated with optimism robustness. We predicted optimistic expectancies to trigger orientation of attention to confirmatory evidence in the environment (i.e. rewards that justify the initial optimism) and to neglect information that is at odds with them. Hypotheses regarding anatomical associations of optimism robustness were inspired by a recent neurophysiological model on the optimism-attention interplay (Kress and Aue, 2017), on one hand, and—due to missing precedence in the structural area—related fMRI research (Singh *et al.*, 2020), on the other hand. Accordingly, gray matter volume (GMV) in the salience and executive control networks, as well as in visual areas, was predicted to play a fundamental role in optimism robustness. Because of the proposed intertwinement of optimism robustness and belief updating asymmetry, additional hypotheses may be derived from voxel-based morphometry (VBM) research on belief updating. Specifically, Chowdhury and colleagues (Chowdhury *et al.*, 2014) investigated GMV in older and younger participants and found that increased belief updating asymmetry in older adults arose because of their limited capacity to integrate undesirable information into their expectancies. Moreover, the strength of such reduced capacity to adjust expectancies following undesirable information in older adults correlated positively with GMV in the dACC.

Whether GMV in these areas plays a role in optimism robustness remains to be determined. Hence, the current study addressed two major questions. First, we wanted to examine whether individuals who are particularly sensitive to optimistic rather than pessimistic cues (and thus present greater optimism robustness) differ in brain anatomy from those who are less sensitive to these cues. To this aim, we performed a whole-brain VBM analysis to investigate the association of GMV with optimism robustness (derived from the participants’ RTs). Second, as little is known about the (potential) overlap of more stable structural and more fluctuating functional aspects in the brain [even outside the area of optimism; (Segall *et al.*, 2012; Sui *et al.*, 2014; Calhoun and Sui, 2016)], we tested whether identified structural correlates of optimism robustness involve specific functional aspects. Anatomical characteristics are the basis for functional changes and interregional communication, and so brain anatomy can be expected to support or complement the observed functional changes. It is hence worthwhile exploring the association of structure and function. Therefore, we also examined whether neural activity in the areas identified by the structural VBM analysis was related to optimism robustness.

To investigate these aims, we examined the gray matter (GM) characteristics of a sample that we previously examined for fMRI and behavioral optimism robustness indexes (Singh *et al.*, 2020). The participants’ task in this study was to detect two different letters (one symbolizing monetary gain and the other monetary loss) as rapidly and as accurately as possible in a visual search matrix. Prior to each onset of a visual search matrix, different verbal cues informed the participants about their chances to gain (optimistic expectancy) or lose (pessimistic expectancy) money. The target presented could thus be either congruent or incongruent with these induced expectancies.

## Materials and methods

### Participants

Participants were 50 (19 male) healthy students of the University of Bern, aged 18–39 years (*M* = 25.06 years, s.d. = 4.68 years). They had normal or corrected-to-normal vision. Exclusion criteria comprised neurological and mental disorders, MRI contraindications, use of psychoactive substances, color blindness [assessed with Ishihara plates; (Ishihara, 1987)] and left handedness. As a group, participants taking part in the present study were characterized by slight dispositional optimism [Life Orientation Test-Revised (Scheier *et al.*, 1994); *M* = 22.56, s.d. = 3.78, on a scale from 7 to 35], high satisfaction with their lives [Satisfaction With Life Scale (Diener *et al.*, 1985); *M* = 26.16, s.d. = 6.21, on a scale from 5 to 35], low trait anxiety [State-Trait Anxiety Inventory (Scheier *et al.*, 1994); *M* = 36.02, s.d. = 7.60, on a scale from 20 to 80] and absence of depression [Beck Depression Inventory-II (Beck *et al.*, 1996); *M* = 5.08. s.d. = 4.31, on a scale from 0 to 63].

Participants were informed that they would be able to end the experiment at any time and gave written informed consent. All procedures met the guidelines of the Declaration of Helsinki and were approved by the Swiss Ethics Committee on research involving humans in the canton Bern, Switzerland (https://swissethics.ch/en/ethikkommissionen). Participation in the study was reimbursed with either course credits or 25 Swiss francs/h. In addition, participants received an extra 5 Swiss francs, representing their gain from the gambling task (see Procedure for details).

### Experimental paradigm

**Fig. 1.**
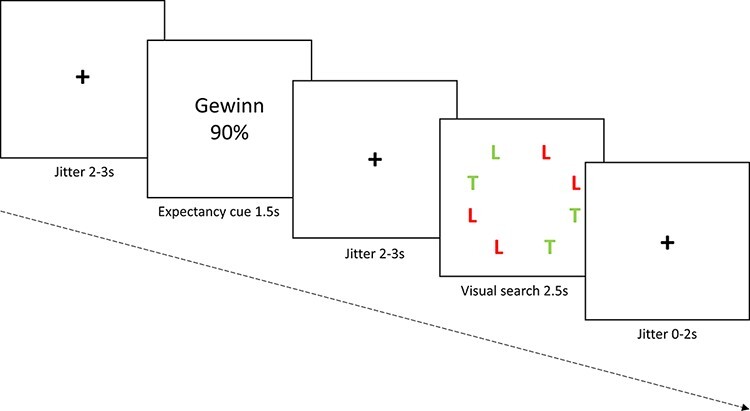
Schematic representation of an experimental trial. At the beginning of each trial, a fixation cross was presented for 2–3 s. Next, participants saw a cue that induced an optimistic expectancy (gain 90%; ‘Gewinn 90%’ in German), a pessimistic expectancy (loss 90%) or an ambiguous expectancy (gain loss 50% and loss gain 50%). This was followed by another fixation cross of 2–3 s and a visual search task (searching for green-colored ‘L’ in the depicted case). Participants had 2.5 s to indicate whether the target had been depicted on the left or the right side of the screen. Finally, another fixation cross was displayed for 0–2 s, ensuring an overall trial length of 10 s.

The experimental paradigm simulated a gambling task consisting of 256 trials in which optimistic, pessimistic and ambiguous expectancies were induced experimentally and gain or loss targets had to be detected in a visual search phase (Figure 1). Expectancies were induced by three different cues. Participants were told that the cue ‘gain 90%’ (‘loss 90%’) signaled a 90% likelihood of a gain (loss) target appearing in a subsequently depicted visual search matrix. As a result of the need to include a sufficiently high number of incongruent trials for data analysis, the actual probability was 67% instead of 90% (leaving the remainder of the gain 90% and loss 90% trials for incongruent cue-target combinations). Because the majority of outcomes were congruent with the prior presented cues, the cues maintained a significant degree of predictability. To minimize distrust in the cues, we further told our participants that the computer randomly selected a target from a pool of 100 targets [comprising 90 gain (loss) and 10 loss (gain) targets for the 90% gain (loss) cues] and that, for this reason, their personal expectancy value might differ from the average value displayed by the cues. The third cue was an ambiguous condition with maximum uncertainty and was included for reasons unconnected to the current manuscript. Because this cue differed in terms of valence, predictability and complexity from the two other cues, it will not be considered here.

Stimuli presented in the visual search phase comprised eight green and red ‘L’s and ‘T’s that were displayed on a circle. The target stimuli were a green ‘L’ (representing monetary gain for one half of the participants and loss for the other half) and a red ‘T’ (reverse representation). Any target stimulus was displayed with equal frequency in any of the eight locations on the circle.

### Procedure

Upon their arrival, participants signed a written informed consent form. They then read the experimental instructions that described the study as a gambling task and performed six practice trials before being comfortably positioned in an MRI scanner (3 Tesla MAGNETOM Prisma Scanner; Siemens, Erlangen, Germany). A projector based on Liquid Crystal Displays (PT-L711E, Panasonic, Kadoma, Japan) enabled the visual projection of the stimuli onto a screen in front of the scanner, which in turn was viewed through a mirror mounted to the scanner’s head coil. The experiment was programed with e-prime 2.0 Professional (Psychology Software Tools, Sharpsburg, PA, USA), on the basis of a similar task used to examine the influence of expectancy variations on attention in the negative affect domain (Aue *et al.*, 2013a, 2016, 2019).

At the beginning of each trial, participants were told that their chance of winning or losing would be 50 or 90%, as manipulated by the visual cues presented (see Experimental Paradigm for further details and Figure 1 for a schematic representation of an experimental trial). Afterward, they had to search for a target stimulus (e.g. green L or red T) in a visual search array containing seven distractors (red Ls and green Ts) in addition to the target. For half of the participants, 25 cents was added to their starting amount of 5 Swiss francs when they saw a green L and 25 cents was subtracted when they saw a red T; for the other half, it was the reverse. The participants’ task was to indicate as quickly and accurately as possible whether the target stimulus had been presented on the left or on the right side of the screen by choosing to press one of two different buttons of a button box that was connected to a response box outside the scanner (Lumina LP400, Cedrus Corporation, San Pedro, CA). Letting participants indicate the part of the screen in which the deviant stimulus is shown (rather than whether the deviant target signals a gain or a loss) rules out the explanation that motor processes (i.e. response preparation) influence the relationship between expectancies and attention.

The task consisted of 256 experimental trials, of which 128 were congruent (64 trials with gain targets preceded by the gain 90% cue; 64 trials with loss targets preceded by the loss 90% cue), 64 were incongruent (32 trials with gain targets preceded by the loss 90% cue; 32 trials with loss targets preceded by the gain 90% cue), and 64 were ambiguous (32 trials with gain targets preceded by the ambiguous gain loss 50% cue; 32 trials with loss targets preceded by the gain loss 50% cue). These trials were presented in four sessions of 64 trials each, allowing for short pauses between sessions. The frequencies of specific cue-target combinations were comparable across sessions. Because of the identical numbers of presentations of gain and loss targets in the experiment, our participants gained and lost 32 Swiss francs and ended up with their starting amount of 5 Swiss francs. In a last step, our participants completed different questionnaires (e.g. regarding affect and personality characteristics) and were subsequently debriefed and paid.

### Structural and functional MRI acquisition

All participants had undergone an MRI, and data were acquired with a 3 Tesla Siemens scanner (MAGNETOM Prisma, Erlangen, Germany) via a 64-channel head coil at the Inselspital, University Hospitals Bern, Switzerland. The structural MRI (sMRI) sequence consisted of a 3D magnetization-prepared rapid gradient-echo sequence. The sequence parameters included repetition time (TR) = 2300 ms, echo time (TE) = 2.98 ms, inversion time (TI) = 900 ms, flip angle = 9°, and matrix size = 160 × 256 × 256, with isotropic voxel resolution = 1 mm^3^. Functional volume registration relied on a T2*-weighted multiband echo-planar imaging (EPI) sequence with a 48-slice whole-brain coverage (slice thickness = 2 mm, 0.5 mm gap, interleaved slice order, TR = 1000 ms, TE = 30 ms, flip angle = 80°, field of view = 192 × 192 mm, matrix size = 96 × 96, voxel size = 2 × 2 × 2.5 mm, PAT mode GRAPPA, acceleration factor 2, multiband factor = 3).

### Data analysis

#### Behavioral data analysis.

Attention orientation represented the dependent variable and was assessed by measuring RTs to the target stimuli in the visual search matrix. Only correct responses (∼92.6% of all responses) were considered. We expected that our participants’ RTs would reveal optimism robustness, which implies that optimistic (i.e. gain) expectancies are more effective in guiding visual attention than pessimistic (i.e. loss) expectancies are. The more effective a cue is, the more strongly it should affect the participants’ behavior in congruent (speeded responding) and incongruent (slowed responding) trials. Accordingly, we predicted a greater difference in RTs between gain and loss trials that were preceded by optimistic (gain 90%) cues than between gain and loss trials that were preceded by pessimistic (loss 90%) cues.

To test for the existence of optimism robustness in our behavioral data, we calculated differences in average RTs between incongruent and congruent experimental trials separately for optimistic (Diff_OptimisticCue_ = RT_OptimisticCue_LossTarget − RT_OptimisticCue_GainTarget) and pessimistic (Diff_PessimisticCue_ = RT_PessimisticCue_GainTarget − RT_PessimisticCue_LossTarget) cues. Support for the optimism robustness hypothesis would be revealed if Diff_OptimisticCue_ > Diff_PessimisticCue_. The hypothesis was tested with a pairwise *t* test. The alpha level of our statistical tests was set to 0.05 (one-tailed) and the reported effect size was Cohen’s *d*, denoted by *d*. In addition, we performed an analysis of variance on our RT data (see Supplementary Analysis).

#### sMRI data analysis: VBM.

All data analysis was performed with SPM12 (www.fil.ion.ucl.ac.uk) via a MATLAB 2017b environment (developed by The MathWorks, Inc.). We used UBELIX (www.ubelix.ch), the cluster services from the University of Bern, Switzerland, for parallel data processing. At first, raw MRI images of the participants (DICOM) were converted into NIfTI format. This was followed by a visual inspection of the images to check for severe motion artifacts and then manually performed AC–PC alignment (anterior–posterior commissure). None of the images suffered any major motion artifacts that might hamper the successive processes. Native T1-weighted images were further segmented into different brain tissue classes, including GM, white matter (WM) and cerebrospinal fluid (CSF) through unified segmentation (Ashburner and Friston, 2005). The GM and WM images were used to create a study-specific brain template in Montreal Neurological Institute (MNI) space by using diffeomorphic anatomical registration through exponential Lie algebra or DARTEL toolbox (Ashburner, 2007). Subsequently, native GM images were normalized to the MNI space with a voxel resolution of 1 mm^3^, while preserving for GM tissue volume in each voxel (modulated). Finally, normalized modulated GM images were smoothed by using a Gaussian kernel of [2 2 2] mm full width at half maximum (FWHM), resulting in an overall smoothing of [4.5 4.3 4.4] mm.

For statistical analysis using general linear models, we performed a multiple regression analysis with six regressors (RT_OptimisticCue_GainTarget, RT_OptimisticCue_LossTarget, RT_PessimisticCue_GainTarget, RT_PessimisticCue_LossTarget, RT_AmbiguousCue_GainTarget and RT_AmbiguousCue_LossTarget) and three covariates of no interest [age, sex and total intracranial volume (TIV = GM_volume_ + WM_volume_ + CSF_volume_)]. Our primary contrast of interest for the VBM analysis included optimism robustness ([RT_OptimisticCue_LossTarget − RT_OptimisticCue_GainTarget] − [RT_PessimisticCue_GainTarget − RT_PessimisticCue_LossTarget] or simply Diff_OptimisticCue_ − Diff_PessimisticCue_). We incorporated a GM binary mask as an explicit mask, so as to confine the findings to this brain tissue. All statistical models were estimated, and results were obtained for both positive and negative correlations at a voxel-level of *P* < 0.005 and a cluster level of *P* < 0.05 family-wise error corrected. This led to a minimum suprathreshold cluster size of *k* = 322 voxels. We used the Automated Anatomical Labeling atlas (Tzourio-Mazoyer *et al.*, 2002) to quantify the suprathresholded findings and named it with its anatomical location(s).

To further investigate the origins of the identified GMV-behavior associations related to optimism robustness, we conducted post hoc region of interest (ROI) analyses. Specifically, we extracted GMV for all clusters revealed and performed Pearson product moment correlation coefficients (controlled for TIV) with the substituents of Diff_OptimisticCue_, namely, (i) RT_OptimisticCue_LossTarget and (ii) RT_OptimisticCue_GainTarget. Similarly, we determined Pearson correlations with the substituents of Diff_PessimisticCue_, namely, (iii) RT_PessimisticCue_GainTarget and (iv) RT_PessimisticCue_LossTarget. In addition, we performed sub-VBM analyses for (combinations) of these constituents of optimism robustness (cf. Supplementary Table S1).

#### Link of sMRI results with fMRI neural activity scores.

fMRI data preprocessing was also performed with SPM 12 and comprised slice-time correction (middle slice as reference), unwarping and spatial realignment (fourth-degree b-spline interpolation). Retrospective noise correction with the Functional Image Artifact Correction Heuristic Package [FIACH; (Tierney *et al.*, 2016)] was performed in R (Team, 2013). FIACH also permitted the calculation of six principal components of physiological noise (likely related to cardiac and respiratory responding). In a next step, the functional data were co-registered to the individual anatomical images and, then, normalized to the standard space of the MNI EPI template and spatially smoothed with an isotropic three-dimensional Gaussian filter with an FWHM of 6 mm.

In our statistical analyses, we applied the general linear model implemented in SPM 12 and modeled event-related percent signal changes (PSCs) separately for each participant. The first-level model included the following regressors: OptimisticCue, PessimisticCue, AmbiguousCue (expectancy phase; duration: 0 s); OptimisticCue_GainTarget; OptimisticCue_LossTarget; PessimisticCue_LossTarget; PessimisticCue_GainTarget; Ambi-guousCue_GainTarget and AmbiguousCue_LossTarget (target phase; duration: 0 s). The model also accounted for RT influences on neural activity: For each of the six target phase regressors, participants’ standardized RTs were entered as parametric modulators of the hemodynamic response. In addition, the first-level model included one regressor for participants’ errors, six movement parameters of the realignment procedure, six physiological noise parameters identified by FIACH (all regressors of no interest) and a constant covariate representing the session-specific mean over scans. The model further comprised a high-pass filter of 128 s to remove low-frequency drift of the scanner and first-order auto-regressive corrections for auto-correlation between scans.

To investigate whether GMV correlates of optimism robustness involve functional aspects, we extracted the fMRI neural activity (in PSC) scores for the clusters we identified in the VBM analysis for optimism robustness. Analogous to the behavioral data, we calculated Diff_OptimisticCue_ (PSC_OptimisticCue_LossTarget − PSC_OptimisticCue_GainTarget) and Diff_PessimisticCue_ (PSC_PessimisticCue_GainTarget − PSC_PessimisticCue_LossTarget). Extracted neural activity scores for Diff_OptimisticCue_ − Diff_PessimisticCue_ (average across all voxels constituting the clusters identified in our VBM analysis) in each participant were correlated with RT scores for optimism robustness by using Pearson product moment correlation coefficients. In addition, we extracted the four task-related neural activity scores (PSC_OptimisticCue_LossTarget, PSC_OptimisticCue_GainTarget, PSC_PessimisticCue_GainTarget, PSC_PessimisticCue_LossTarget) making up the optimism robustness score and performed a paired *t* test contrasting Diff_OptimisticCue_ with Diff_PessimisticCue_ within participants. By this means, we tested whether there were any task-related within-participant fMRI effects related to optimism robustness (that are not necessarily correlated with the participants’ RTs).

## Results

### Behavioral data

**Fig. 2.**
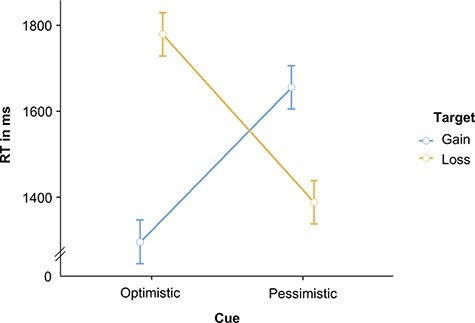
Depiction of RTs in the current experiment. Error bars depict standard errors. Optimistic cue = gain 90%, pessimistic cue = loss 90%.

Consistent with our optimism robustness hypothesis, optimistic expectancies more strongly affected our participants’ RTs in the visual search phase than did pessimistic expectancies—indexed by a greater difference between incongruent and congruent trials for gain cues (Diff_OptimisticCue_: *M* = 483 ms, *SE* = 52 ms) than for loss cues (Diff_PessimisticCue_: *M* = 267 ms, *SE* = 60 ms), *t*(49) = 2.76, *P* = 0.004, *d* = 0.39 (Figure 2). Supplementary Analysis 1 displays, in addition, the results of an analysis of variance conducted on our participants’ RTs to provide more detailed information on RTs related to the individual task conditions.

### sMRI data: VBM

#### Whole-brain VBM: association of GMV with optimism robustness expressed in RTs.

**Fig. 3.**
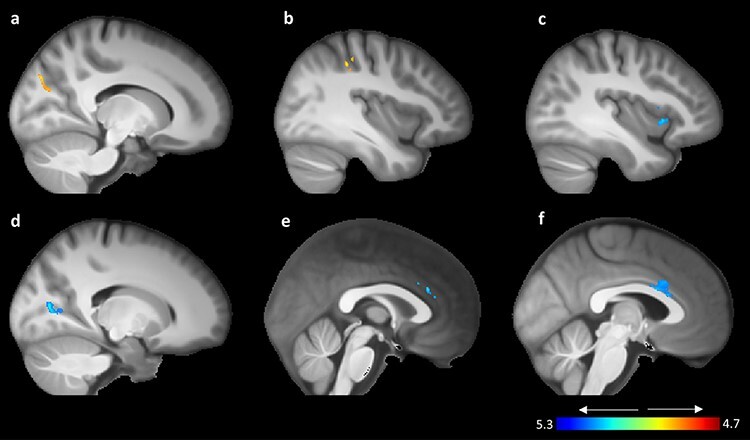
VBM GMV findings for positive (red–yellow color bar) and negative (blue–light blue color bar). All findings are overlaid on a normalized averaged skull-extracted brain template created from the participant cohort and presented from a sagittal view. (a) Right medial visual association area, (b) right intraparietal sulcus, (c) right insula, (d) right primary visual cortex, (e and f) dACC.

We identified two clusters located in the medial visual association area and the intraparietal sulcus in which GMV was positively associated with optimism robustness (Table 1, upper part, and Figure 3A–B). Post hoc ROI analyses and sub-VBM analyses for the specific sub-conditions that, together, form the phenomenon of optimism robustness (Supplementary Table S1) were subsequently conducted to determine the origins of the effects observed. These additional analyses revealed that the effects arose because of differences in RTs between incongruent and congruent targets following optimistic cues. Faster responding to gains following optimistic expectancies, in particular, were associated with higher GMV in the medial visual association area and the intraparietal sulcus.

**Table 1.** VBM GMV findings: association with optimism robustness

T-max	MNI x y z			Cluster size (k)	Anatomical region	Correlation with RT_OptimisticCue_LossTarget r (*P*)	Correlation with RT_OptimisticCue_GainTarget r (*P*)	Correlation with RT_PessimisticCue_GainTarget r (*P*)	Correlation with RT_PessimisticCue_LossTarget r (*P*)	Association with neural activity r (*P*)
Positive correlation between GMV and optimism robustness										
4.61	14	−79	33	384	R Medial visual association area (extrastriate cortex)	0.12 (0.411)	**−0.35 (0.011)**	−0.12 (0.409)	−0.09 (0.519)	−0.08 (0.585)
4.25	38	−36	44	364	R Intraparietal sulcus	−0.06 (0.555)	**−0.47 (0.001)**	−0.23 (0.113)	−0.20 (0.168)	−0.02 (0.869)
Negative correlation between GMV and optimism robustness										
4.36	37	12	8	1153	R Insula	**−0.40 (0.004)**	**−0.30 (0.040)**	−0.16 (0.260)	**−0.39 (0.006)**	−0.13 (0.365)
4.41	−38	15	−2	765	L Insula	**−0.48 (0.0004)**	**−0.31 (0.031)**	−0.25 (0.085)	**−0.38 (0.006)**	−0.11 (0.439)
5.31	17	−72	11	1211	R Primary visual cortex	0.01 (0.959)	**0.42 (0.003)**	0.26 (0.067)	0.19 (0.197)	−0.10 (0.502)
4.53	1	29	25	413	dACC	−0.27 (0.062)	−0.12 (0.393)	0.01 (0.952)	−0.23 (0.102)	−0.12 (0.414)
4.04	−3	11	25	391	dACC	**−0.43 (0.002)**	−0.12 (0.393)	0.05 (0.706)	**−0.37 (0.008)**	−0.09 (0.521)

Bold: *P* < 0.05. Right column: Pearson product moment correlation coefficients for the association between optimism robustness (as revealed in RTs) and average neural activity within the areas identified by our VBM analysis. L = left, R = right.

Moreover, GMV in five additional clusters revealed negative associations with optimism robustness (Table 1, lower part, and Figure 3C–F). Two of those clusters centered in the right and left anterior insula (the latter extending substantially into the putamen), two others in the dACC and one in the right primary visual cortex. Post hoc analyses for the two insula and the two dACC ROIs showed that, descriptively, higher GMV in these areas was associated with faster response, especially for the loss targets. Supplementary Table S1 reveals that the difference in RTs between gain and loss targets following optimistic cues was significantly associated with GMV in the two insula clusters, whereas this was not the case when targets had been preceded by pessimistic cues. In contrast, post hoc VBM analyses conducted for the dACC did not yield any significant effect. Finally, a higher GMV in the right primary visual cortex was associated with differential responding to gains and losses following optimistic cues (Supplementary Table S1). Faster responding to gains following optimistic expectancies, in particular, was associated with lower GMV in this area.

#### Link with fMRI neural activity scores.

To test whether areas in which GMV varied as a function of optimism robustness also involved functional correlates with optimism robustness, we calculated Pearson product moment correlation coefficients between optimism robustness (measured by our participants’ RTs) and neural activity in the clusters that we had identified in our VBM analysis. Table 1 (right column) shows that none of the regions displayed both anatomical and functional associations with optimism robustness.

We further tested whether there were any task-related within-participant fMRI effects related to optimism robustness. Regions identified by our VBM approach did not reveal any neural activity difference between congruency effects related to optimistic *vs* pessimistic cues (contrast of PSC_OptimisticCue_LossTarget − PSC_OptimisticCue_GainTarget with PSC_PessimisticCue_GainTarget − PSC_PessimisticCue_LossTarget), as calculated within participants (Supplementary Table S2). Correspondingly, overall, there was no indication that the neural activity in the regions identified in our VBM analysis was related to optimism robustness.

## Discussion

In our model on optimism-attention interplay (Kress and Aue, 2017), we postulate that optimistic expectancies drive attention selectively to rewarding evidence in the environment. Previous research (Kress *et al.*, 2018; Singh *et al.*, 2020) has shown that such priming effects of expectancies on attention are generally stronger for optimistic than pessimistic content. This phenomenon, according to which individuals are more sensitive to optimistic than to pessimistic cues (Kress *et al.*, 2018; Singh *et al.*, 2020), has been termed optimism robustness. With the current study, we aimed at identifying brain structural correlates of optimism robustness. To address this aim, we conducted a whole-brain VBM analysis, regressing GMV by optimism robustness in a visual search task that was developed to measure selective visual attention. From our model (Kress and Aue, 2017) and earlier functional findings (Singh *et al.*, 2020), we predicted that GMV in the salience and executive control networks, as well as in visual areas, would play a fundamental role in optimism robustness.

In line with this hypothesis, our VBM analysis yielded two clusters—located in the medial visual association area and the intraparietal sulcus—in which GMV was positively correlated with optimism robustness. Specifically, GMV in both regions was negatively associated with RT for gain targets following optimistic cues; thus, individuals who displayed particularly fast responses to gains that confirmed their initial optimistic expectancies had a higher volume in the medial visual association area and the intraparietal sulcus. Both regions have been shown to be key to selective visual attention (Booth *et al.*, 2003; Behrmann *et al.*, 2004; Hahn *et al.*, 2008; Aue *et al.*, 2013b; Salo *et al.*, 2017). Together, these data corroborate that optimism robustness assessed in our study reflects enhanced selective attention. Such an interpretation is supported by earlier eye tracking data (Kress *et al.*, 2018), which revealed that (i) individuals more strongly orient toward evidence that confirms their initial optimism than to evidence that confirms their initial pessimism, and (ii) they in addition maintain their attention more strongly on evidence that justifies their initial optimism.

We also observed, consistent with this interpretation of selective visual attention, that the volume of the primary visual cortex correlated negatively with optimism robustness, which again was mostly due to the optimism cue–gain target condition subcomponent. In other words, the larger the optimism robustness phenomenon, the smaller the volume of the primary visual cortex. This region has been linked with, among other things, the generation of mental images: a larger visual cortex thickness predicts mental imagery precision [e.g. (Bergmann *et al.*, 2016b)], and a larger visual cortex surface predicts visual perception acuity [e.g. (Song *et al.*, 2015)]. Interestingly, the interindividual variability in the size of the primary visual cortex reflects a trade-off between sensitivity to visual details and susceptibility to visual context in the perception of the same visual feature (Song *et al.*, 2013): A larger primary visual cortex ensures local, detail-oriented perception (e.g. high visual discrimination of fine details), whereas a smaller cortex (as in our finding for optimism robustness) predisposes to global, context-oriented perception, making individuals more prone to visual illusions and biases [e.g. (Muckli, 2010; Patten *et al.*, 2017)], likely via low weighting or ignorance of prediction errors (Clark, 2013; Aitken *et al.*, 2020). Specifically, context-oriented perception exaggerated by a small visual cortex prioritizes, among other things, prior expectations of the incoming stimulus (Khan and Hofer, 2018). When certainty about an upcoming stimulus is high (e.g. prior expectation of a gain in an optimistically cued trial), the magnitude of the prediction error is minimal when the actual stimulus is identical to the expectation (i.e. gain), but the magnitude increases when the stimulus deviates from the expectation (i.e. loss) (Den Ouden *et al.*, 2012). A small primary visual cortex might prevent such details (i.e. predicting errors) from being integrated into future expectancies. Correspondingly, a small primary visual cortex may not generate the optimism robustness phenomenon per se, but it likely exaggerates it by overweighing an already influential prior expectation.

Moreover, other anatomical findings for the primary visual cortex suggest that visual working memory storage varies as a positive function of its GMV [e.g. (Bergmann *et al.*, 2016a)]. One may therefore speculate that those individuals who displayed particularly strong optimism robustness in our study may have been characterized by reduced working memory capacity or engagement, thereby allowing for a greater shift toward an optimistic outlook. In particular, reduced involvement of working memory could facilitate the neglect of pessimistic cues, which is consistent with our observation of positive correlations between GMV in the primary visual cortex and RTs in both pessimistic cue conditions (Table 1). Consistent with such an interpretation, the calcarine gyrus has also been reported to be involved in belief updating (Kuzmanovic *et al.*, 2016). The study authors reported that greater updating was affiliated with decreasing activity in the calcarine gyrus, especially in response to negative feedback (i.e. feedback suggesting that one should shift one’s future expectancies to the pessimistic direction).

In line with our hypotheses based on the optimism-attention interplay model (Kress and Aue, 2017), we further found two areas of the salience network (Doucet *et al.*, 2019) to be implicated in optimism robustness, namely, two clusters in each of the dorsal anterior insula (in one case extending into the putamen) and the dACC. However, while one would be inclined to claim greater optimism robustness for highly salient experiences (supposedly indexed by greater GMV in the salience network), we observed the opposite [cf., (Singh *et al.*, 2020)]: GMV in the anterior insula and dACC correlated negatively with the extent of optimism robustness displayed. Post hoc analyses revealed that, descriptively, higher GMV in these regions corresponded with faster responding, especially in the loss target conditions. Sub-VBM analyses revealed GMV in the insula to vary significantly as a function of differences for gain *vs* loss targets following optimistic expectancies (but not pessimistic cues). Detecting threat and punishment in the environment is one of the putative functions of the salience network (McMenamin *et al.*, 2014), and the detection and perception of threat and punishment in the environment correlate with larger volumes (Carlson *et al.*, 2012) and higher activation of the ACC (Bishop *et al.*, 2004). A lower sensitivity of the salience network to threat and punishment, in particular while having optimistic expectancies, could prompt individuals to become more optimistically biased, and a larger GMV within the salience network might counteract optimism robustness.

Consistent with such an interpretation, research on a highly related concept, comparative optimism (the degree to which we are overly optimistic for ourselves compared with the optimism we display for others), has shown that anterior insular activity indexes the degree to which negative outcomes are considered to apply to the self (Blair *et al.*, 2013). Together with the demonstration of the anterior insula being involved in awareness (in particular of negative information) (Knutson and Greer, 2008; Craig and Craig, 2009), these findings on the insula hence support the idea that optimism robustness varies as a negative function of the degree to which negative future events are considered (Dricu *et al.*, 2020)—especially when being in an optimistic state. Expressed differently, optimism robustness may rely on restricted awareness of undesirable future happenings. Such an interpretation is fully in line with our observation of reduced visual attention (as measured with eye tracking) to undesirable outcomes (Kress *et al.*, 2018) in another sample that faced the exact same experimental paradigm that we used in the current research [see (Peters *et al.*, 2016), for similar observations]. In sum, participants with particularly small-sized key regions within the salience network were prone to display optimism robustness. Our sub-VBM analyses suggest that this effect may, in particular, be attributed to the negative correlation between RTs for the optimistic cue–loss target condition and GMV in the insula (Supplementary Table S1; at the same time, these analyses do not provide information regarding specific origins of the dACC effects).

Together, therefore, our data speak to two major processes being involved in optimism robustness: (i) the capacity to rapidly process and respond to expected rewards (special status of the optimistic cue–gain target condition), which is likely most strongly linked with GMV in areas involved in selective attention and global, context-driven processing, and (ii) the capacity to process unexpected punishments or hindrances (special status of the optimistic cue–loss target condition), which may be best indexed by GMV in brain regions that have been shown to be involved in salience estimations and awareness of negative outcomes.

It may further seem surprising that we did not find any overlap between our structural and functional (Singh *et al.*, 2020) findings. In particular, the previously published functional data revealed increased activity in the salience and executive control networks to correlate with increasing levels of optimism robustness, suggesting that disconfirmed optimistic expectancies were not simply ignored. A possible explanation for this inconsistency is that the structure–function relationship does not necessarily follow a one-to-one mapping, because structures only partially determine the blood-oxygen-level-dependent signal of fMRI (Park and Friston, 2013; Mišić *et al.*, 2016; Batista-García-Ramó and Fernández-Verdecia, 2018). Furthermore, the link between volume and activity is not straightforward (Segall *et al.*, 2012), reflected in the observation that some areas are characterized by positive associations between functional and structural aspects, others by negative associations, and still others by no association and that sMRI and fMRI yield complementary information (Sui *et al.*, 2014; Calhoun and Sui, 2016).

In fact, the extent of neural activity is only one aspect that possibly relates to variations in GMV. Another aspect regards connections with other areas in the brain. For instance, we found that social optimism bias (i.e. the degree to which we are optimistically biased for those we identify with or those we like) varied as a function of the functional connectivity between the visual cortex and the human reward system, although none of these regions displayed isolated activity related to social optimism bias (Aue *et al.*, 2012). Accordingly, future investigations may apply graph theory (Kaiser, 2011; Korgaonkar *et al.*, 2014; Ueda *et al.*, 2018; Pang *et al.*, 2019) to identify WM connections for the different areas identified in our current VBM analyses. Such an approach may reveal important insights into the structural characteristics of optimism robustness. We might, for instance, learn that interregional communication between the key areas identified in our present analyses plays an important role in optimism robustness. Interestingly, cortical–subcortical structural connectivity analyses that identify areas involved in belief updating (Moutsiana *et al.*, 2015) yielded results (i.e. related to insula/putamen) that partially converged with our VBM optimism robustness findings, which may point to an overlap of the two concepts under investigation. The exact degree of overlap remains to be determined by the application of similar measures and analyses to both data sets.

Finally, several specificities of the experimental paradigm used merit consideration. Telling our participants that the optimistic and pessimistic cues would correctly predict the targets on an average of 90% of all cases could potentially be a limitation. In order to ascertain a sufficiently high number of valid incongruent trials (and, simultaneously, an acceptable study length), in reality, the cues correctly predicted the targets in only 67% of all cases. Although this discrepancy may have led to some distrust in the cues, we believe that it does not question the validity of our experimental paradigm because (i) we informed our participants that the computer would randomly select trials from a pool of trials with an overall 90% contingency between optimistic and pessimistic cues, on one hand, and the gain and loss targets, on the other hand; (ii) even with this value being reduced to 67%, the cues still had predictive power; (iii) experimental instructions on proportions can be more efficient in influencing behavior than real proportions (Entel *et al.*, 2014) and, finally, (iv) behavioral data corresponding to the type of paradigm that we used in the current analyses repeatedly revealed that the cues substantially affect attention deployment (Aue *et al.*, 2013a, 2016, 2019; Kress *et al.*, 2018). Together, these considerations strongly argue against our cues having been ineffective (i.e. distrusted). In addition, we may be criticized for not having included an adequate neutral baseline condition. This is because our ambiguous (not considered) third cue condition differed from the optimistic and pessimistic cue conditions in valence, complexity and degree of predictability. Future studies on optimism robustness should add a suitable baseline condition that permits an interpretation of effects in absolute terms (i.e. facilitatory optimistic cue effects and/or suppressive pessimistic cue effects).

## Summary and conclusions

The current study findings demonstrate that greater sensitivity to optimistic than to pessimistic cues in the environment is associated with GMV in the human brain. Our findings further promote the picture of optimism robustness being a result of selective turning toward those pieces of evidence in the world that support one’s positive view of the future. Consequently, structural correlates of optimism robustness may ensure an orientation to rewards in the environment, which stabilizes an optimistic outlook, thereby boosting mental health. Specifically, we found optimism robustness to be related to increased GMV in areas involved in selective attention (the medial visual association area and the intraparietal sulcus), reflective of the strong intertwinement of optimistic expectancies and attention deployment (Kress and Aue, 2017). In addition, GMV in the primary visual cortex diminished with increasing levels of optimism robustness, which is in line with (i) the idea of a trade-off between sensitivity to details and susceptibility to context in perception, the latter predisposing for positive cognitive biases (such as optimism robustness), and (ii) the interpretation of reduced working memory being at the basis of optimism robustness. Reduced working memory capacity may permit an individual to rely on default responding, favoring optimistic over pessimistic tendencies. Whether biased memory processes indeed mediate the link between expectancies and attention should be addressed in future research.

All of the aforementioned effects arose because RTs to gains following optimistic cues varied most strongly as a function of GMV. In addition, we observed decreasing GMV in the salience network (namely, the anterior insula and the dACC) with increasing levels of optimism robustness, which arose because GMV in those structures correlated negatively with RTs for the processing of loss targets. Subanalyses for the insula pointed to a specific status of the optimistic cue–loss target condition, which describes the need to process unexpected loss and correct overly optimistic expectancies. In sum, therefore, the strongest determinants of the effects we observed were related to (i) the ability to process and respond to expected gains (condition optimistic cue—gain target; attention areas) and (ii) the capability to process and respond to unexpected losses (condition optimistic cue—loss target; salience and awareness areas).

Future research needs to address how malleable the association between optimism robustness and GMV is. For instance, individuals with depression are typically characterized by reduced optimism (Carver and Gaines, 1987; Giltay *et al.*, 2006; Peleg *et al.*, 2009), and diverse types of training have been developed to strengthen positive outlooks (Fresco *et al.*, 2009; Sharot *et al.*, 2012; Reivich *et al.*, 2013; Seligman *et al.*, 2007; Kress and Aue, 2019). It will be compelling to examine whether GMV in the areas that we identified here changes as training-induced optimism robustness increases, particularly so in clinical populations (e.g. individuals with depression). Neuroplasticity after training has been shown in the area of (negative) attention bias (Aday and Carlson, 2017), demonstrating that structural correlates of biased stimulus processing do not condemn people to see the world around them in either negative or positive hues.

As compromised optimistic outlooks constitute a key characteristic of depression, the identification of morphological correlates of optimism robustness and its subcomponents in healthy individuals may provide important information for potential toeholds in the therapeutical context. Because our study revealed that structural correlates of optimism robustness rely on two subcomponents of optimism robustness (capacity to process and respond to expected rewards and capacity to process and respond to unexpected losses after initial optimism), it will be interesting to investigate whether training that targets specific brain structures will have selective effects on these subcomponents. Ultimately, such an approach might help in the development of individualized training, the goal being to modify critical subcomponents of the optimism robustness concept. Hence, individuals who display troubles in the pursuit of expected gains (compared with their peers) would require different training than would individuals whose problems originate in the discouragement arising from problematic processing of unexpected losses.

## Supplementary Material

nsab075_Supp

## Data Availability

Under the Swiss guidelines of data protection (Ordinance HFV Art. 5), the data sets generated and analyzed during the current study can be made available from the corresponding author on a case-by-case basis.
