# Supplementary material for: Enhanced sensitivity to optimistic cues is manifested in brain structure: a voxel-based morphometry study
Source: Soc Cogn Affect Neurosci. 2021 Jun 29;16(11):1170–81. doi: 10.1093/scan/nsab075 (PMC8599192; doi:10.1093/scan/nsab075)
Supplement: nsab075_Supp [file nsab075_supp.zip › scan-21-037-File006.docx]

**Supplementary Analysis 1: Repeated-measures analysis of variance on reaction times.**

For completeness, we also conducted an analysis of variance with the within-participant factors *Cue* (optimistic, pessimistic) and *Target* (gain, loss) on the participants’ RTs in the visual search task. The analysis performed yielded a main effect of *Target*, *F*(1, 49) = 7.62, *p* = 0.008, *η^2^_p_* = 0.14, which was qualified by the significant interaction *Cue* × *Target*, *F*(1, 49) = 85.20, *p* < 0.001, *η^2^_p_* = 0.64 (Figure 2). Post hoc Tukey tests for this interaction revealed significant differences (*p* < 0.020) for all pairwise comparisons except for the one related to the difference between the two congruent conditions RT_OptimisticCue_GainTarget vs. RT_PessimisticCue_LossTarget (*p* = .121). The main effect of *Cue* was not significant, *F*(1, 49) = 1.65, *p* = 0.205, *η^2^_p_* = 0.03.
