# Supplementary material for: Enhanced sensitivity to optimistic cues is manifested in brain structure: a voxel-based morphometry study
Source: Soc Cogn Affect Neurosci. 2021 Jun 29;16(11):1170–81. doi: 10.1093/scan/nsab075 (PMC8599192; doi:10.1093/scan/nsab075)
Supplement: nsab075_Supp [file nsab075_supp.zip › scan-21-037-File007.docx]

**Supplementary Table S1**. Secondary VBM gray matter volume findings.

| **Contrast** | **T-max** | **MNI**  **x y z** | | | **Cluster size (k)** | **Anatomical region** |
| --- | --- | --- | --- | --- | --- | --- |
| Diff_OptimisticCue_ | 4.86 | 23 | -67 | 17 | 1107 | R Medial primary and association visual cortex |
| Diff_OptimisticCue_ | 5.73 | 34 | -39 | 43 | 331 | R Intraparietal sulcus |
| negative Diff_OptimisticCue_ | 4.35 | -39 | 15 | -2 | 1226 | L Insula extending into the frontal operculum |
| negative Diff_OptimisticCue_ | 3.99 | 37 | 12 | 8 | 416 | R Insula extending into the frontal operculum |
| negative Diff_OptimisticCue_ | 3.74 | -23 | -56 | 5 | 648 | L Secondary visual cortex |
| negative Diff_OptimisticCue_ | 4.54 | 19 | -72 | 6 | 1156 | R Primary visual cortex |
| negative Diff_OptimisticCue_ | 4.63 | 39 | -52 | 40 | 470 | R Angular gyrus |
| negative Diff_PessimisticCue_ | 5.90 | -17 | 40 | -16 | 330 | L Orbitofrontal cortex |
|  |  |  |  |  |  |  |
| PessimisticCue_LossTarget | 4.54 | -7 | 13 | -9 | 633 | L Caudate/nucleus accumbens |
| PessimisticCue_GainTarget | 3.97 | -45 | -28 | 34 | 356 | L Postcentral gyrus |
| Negative PessimisticCue_GainTarget | 4.43 | 9 | 51 | 23 | 341 | Dorsomedial prefrontal cortex |
| Negative OptimisticCue_GainTarget | 5.14 | 21 | -59 | 16 | 1445 | R Visual association area |
| Negative OptimisticCue_GainTarget | 5.48 | 36 | -38 | 44 | 550 | R Intraparietal sulcus |
| Negative OptimisticCue_LossTarget | 4.06 | 38 | -6 | -3 | 376 | R Insula extending into the frontal operculum |
| Negative OptimisticCue_LossTarget | 3.86 | -34 | 9 | 5 | 352 | L Insula extending into the frontal operculum |
|  |  |  |  |  |  |  |
| LossTarget_Expected-Unexpected | 4.31 | -7 | 13 | -9 | 786 | L Caudate/nucleus accumbens |
| LossTarget_Unexpected-Expected | 4.82 | 52 | -26 | 14 | 331 | R Posterior superior sulcus |
| GainTarget_Unexpected-Expected | 4.73 | 21 | -57 | 15 | 322 | R Visual association area |

Diff_OptimisticCue_ = (RT_OptimisticCue_LossTarget - RT_OptimisticCue_GainTarget); Diff_PessimisticCue_ = (RT_PessimisticCue_GainTarget - RT_PessimisticCue_LossTarget). L = left, R = right.
