# Supplementary material for: Enhanced sensitivity to optimistic cues is manifested in brain structure: a voxel-based morphometry study
Source: Soc Cogn Affect Neurosci. 2021 Jun 29;16(11):1170–81. doi: 10.1093/scan/nsab075 (PMC8599192; doi:10.1093/scan/nsab075)
Supplement: nsab075_Supp [file nsab075_supp.zip › scan-21-037-File008.docx]

**Supplementary Table S2**. fMRI results for the clusters identified in the VBM analyses.

|  | **Positive optimism robustness** | | **Negative optimism robustness** | | | | |
| --- | --- | --- | --- | --- | --- | --- | --- |
|  | 14 -79 33;  R Medial visual association area | 38 -36 44; R Intraparietal sulcus | 37 12 8;  R Insula | -38 15 -2;  L Insula | 17 -72 11: R Primary visual cortex | 1 29 25; Dorsal anterior cingulate cortex | -3 11 25; Dorsal anterior cingulate cortex |
| Contrast Diff_OptimisticCue_ vs. Diff_PessimisticCue_ |  |  |  |  |  |  |  |
| *t* | -0.914 | -0.555 | -0.891 | -0.739 | -0.927 | -0.548 | -0.390 |
| *p* | 0.365 | 0.581 | 0.377 | 0.464 | 0.358 | 0.586 | 0.698 |
| *d* | -0.129 | -0.079 | -0.126 | -0.105 | -0.131 | -0.078 | -0.055 |

df = 49. L = left; R = right. Coordinates refer to MNI space.
